# Adult hippocampal neurogenesis and its role in Alzheimer's disease

**DOI:** 10.1186/1750-1326-6-85

**Published:** 2011-12-22

**Authors:** Yangling Mu, Fred H Gage

**Affiliations:** 1 Laboratory of Genetics, the Salk Institute for Biological Studies, La Jolla, CA 92037, USA

**Keywords:** Alzheimer's disease, adult neurogenesis, hippocampus, neural stem cell

## Abstract

The hippocampus, a brain area critical for learning and memory, is especially vulnerable to damage at early stages of Alzheimer's disease (AD). Emerging evidence has indicated that altered neurogenesis in the adult hippocampus represents an early critical event in the course of AD. Although causal links have not been established, a variety of key molecules involved in AD pathogenesis have been shown to impact new neuron generation, either positively or negatively. From a functional point of view, hippocampal neurogenesis plays an important role in structural plasticity and network maintenance. Therefore, dysfunctional neurogenesis resulting from early subtle disease manifestations may in turn exacerbate neuronal vulnerability to AD and contribute to memory impairment, whereas enhanced neurogenesis may be a compensatory response and represent an endogenous brain repair mechanism. Here we review recent findings on alterations of neurogenesis associated with pathogenesis of AD, and we discuss the potential of neurogenesis-based diagnostics and therapeutic strategies for AD.

## Introduction

Alzheimer's disease (AD), first described by the German neuropathologist Alois Alzheimer as *Dementia Praecox *in 1907, is an age-related neurodegenerative disease characterized by progressive loss of memory and deterioration of cognitive functions. Individuals with the disorder usually experience difficulties in learning, performance speed, recall accuracy and/or problem solving [1]. The gradual intraneuronal accumulation of neurofibrillary tangles formed as a result of abnormal hyperphosphorylation of cytoskeletal tau protein, extracellular deposition of amyloid-β (Aβ) protein as senile plaques, and massive neuronal death represent important neuropathological hallmarks of AD [2]. These pathologies are evident in specific, vulnerable brain areas and the hippocampus is one of the earliest to be affected [3]. The hippocampus is a mammalian brain structure that lies under the medial temporal lobe, with one on each side of the brain. Although there is a lack of consensus relating to terms describing the hippocampus and its adjacent cortex, the term hippocampus or hippocampal formation generally applies to the dentate gyrus (DG), the hippocampus proper - composed of CA1, CA2 and CA3 fields - and the subiculum. The organization of the hippocampal circuitry has been traditionally characterized as a unidirectional, trisynaptic excitatory pathway (reviewed in [4]). Briefly, the entorhinal cortex (EC) provides the main source of input to the hippocampus through connections to the DG. Information flow then proceeds from DG to CA3 to CA1. In turn, CA1 projects to the subiculum and sends the hippocampal output back to the deep layers of EC. Behavioral studies have long suggested that the hippocampus plays a critical role in learning and memory [5], which depend on functional and structural changes occurring in the hippocampus, such as long-term potentiation (LTP) and synaptic remodeling [6,7]. The discovery of a *de novo *production of neurons in the adult DG has introduced the possibility of a new form of plasticity that could sustain memory processes. A growing body of evidence supports the view that promotion of adult hippocampal neurogenesis improves pattern separation and spatial memory [8,9]. In contrast, a decline in neurogenesis may underlie cognitive impairments associated with aging and disorders such as AD [10,11]. Interestingly, increasing evidence shows that central molecular players in AD influence the generation of new hippocampal neurons, and noteworthy alterations in neurogenesis take place earlier than the onset of hallmark lesions or neuronal loss [12]. Although seemingly contradictory results have been reported in both murine and human studies [13], preserving or potentiating the production of new neurons has been regarded as a potential therapeutic strategy to delay or halt AD-linked cognitive decline. Furthermore, understanding the mechanisms of changes in neurogenesis observed at initial and later stages of AD will contribute to the development of early AD biomarkers and reveal insights into the pathogenesis of AD. In this manuscript, we will summarize current knowledge of the neurogenic roles of molecules whose mutations cause AD, and we will discuss the potential application of newly generated hippocampal neurons in the diagnosis and treatment of AD.

## Neurogenesis in the adult hippocampus

**Figure 1:**
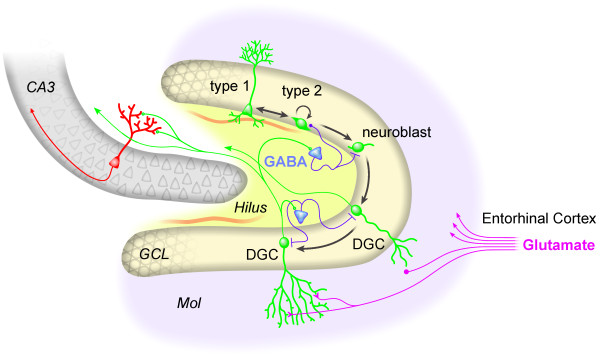
**Neurogenesis in the adult hippocampus**. A population of radial cells in the SGZ corresponds to quiescent NSCs (type 1 cells). They coexist with actively proliferating nonradial NSCs (type 2 cells) that generate both astrocytes and neuroblasts. Neuroblasts migrate into the granule cell layer (GCL) and differentiate into dentate granule cells (DGCs). Newborn DGCs gradually develop elaborate dendritic trees in the molecular layer (Mol) to receive inputs from the EC and project to CA3 pyramidal neurons (red) as well as hilar interneurons (blue).

Continual production of new neurons in the adult hippocampus was first reported by Altman and colleagues [14]. These new neurons originate from self-renewing and multipotent adult neural stem cells (NSCs) residing in the subgranular zone (SGZ) of DG [15]. Two types of NSCs have been identified based on their specific morphologies, proliferative behaviors and expression of unique sets of molecular markers (reviewed in [16]). Type 1 neural progenitor cells (NPCs) have a radial process spanning the entire granule cell layer and ramifying in the inner molecular layer of DG. They are generally identified by specific molecular markers such as GFAP, Sox2 and Nestin. These cells have been hypothesized to be the quiescent stem cells that generate the second type of NSC, the actively self-amplifying, nonradial type 2 cells. These intermediate cells, expressing Sox2, Nestin but not GFAP, subsequently give rise to DCX+ neuroblasts that differentiate into glutamatergic dentate granule cells (DGCs) populating the inner third of the granule cell layer. Young adult rats generate approximately 9,000 new cells in the SGZ each day, with a survival rate of ~50% [17]. The surviving cells send dendrites to the molecular layer of DG to receive inputs from the EC and send axonal projections to the CA3 subfield of the hippocampus to innervate hilar interneurons, mossy cells and CA3 pyramidal cells (Figure 1), thereby integrating into existing neuronal circuits [18,19]. The process of new neuron generation and incorporation involves multiple crucial steps. Many layers of regulation, composed of both intrinsic and extrinsic mechanisms, have been identified for each step. For example, a number of morphogens that are critical during embryonic development of the nervous system, including Notch, Shh, Wnts, and BMPs, are conserved and continue to serve as niche signals to regulate maintenance, activation, and fate choice of adult NSCs. Various neurotransmitter systems, growth factors, neurotrophins, cytokines, and hormones are also major regulators of different phases of adult neurogenesis. Additionally, intrinsic factors such as miRNAs, transcription factors, cell-cycle regulators, and epigenetic factors exhibit cell-autonomous characteristics that provide adult NSCs with the potential to proliferate, differentiate, and survive as newborn neurons. Comprehensive reviews on this subject can be found elsewhere [16,20].

During maturation, newly formed DGCs differ substantially from their older, neighboring counterparts in terms of electrophysiological properties. Typically, they exhibit enhanced synaptic plasticity with both increased amplitude and decreased induction threshold for LTP [21-24]. While the precise functional implications of the continuous production of new neurons with hyperexcitability and enhanced synaptic plasticity are still under intense investigation, it has become increasingly apparent that neurogenesis in the adult DG contributes to various types of hippocampus-dependent learning and memory. The first definitive evidence was provided by the demonstration that a substantial reduction in the number of newly generated DGCs by an antimitotic agent MAM disrupted trace eye-blink conditioning and trace fear conditioning, both of which are regarded to be hippocampus-dependent [25]. A number of subsequent studies utilizing irradiation or genetic modification to eliminate adult neurogenesis in rodent models also observed impaired performance in spatial-navigation learning and long-term spatial memory retention, spatial pattern discrimination, trace conditioning and contextual fear conditioning, clearance of hippocampal memory traces, and reorganization of memory to extrahippocampal substrates (reviewed in [26,27]). Conversely, genetic manipulation and deep brain stimulation that boost neurogenesis have been shown to facilitate pattern separation and water-maze memory, respectively [8,9]. With a combination of theoretical and experimental approaches at cellular, network and system levels, future studies will clarify how addition of a relatively small number of new neurons can modulate global hippocampal or brain function.

## Neurogenesis deficits in AD mouse models

**Figure 2:**
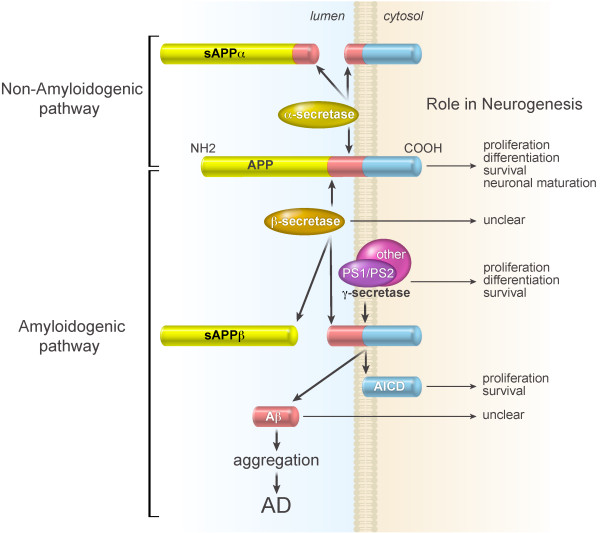
**Molecular basis for AD and interaction with neurogenesis in the adult hippocampus**. In the non-amyloidogenic pathway, APP is processed by α-secretase that cleaves within the Aβ domain. sAPPα and a membrane-bound carboxyl-terminal fragment are generated. In the amyloidogenic pathway, APP is sequentially cleaved by β- and γ-secretases to release the neurotoxic Aβ peptide. β-secretase cleavage of APP forms a secreted ectodomain sAPPβ and a membrane-bound fragment. Cleavage of the latter product generates AICD and Aβ. Suggested functions of relevant signal molecules in adult hippocampal neurogenesis are summarized.

As described in the introduction, senile plaques, a neuropathological hallmark of AD, are made up largely of 38-43 amino acid β-amyloid peptides (Aβ), which are liberated from a larger integral membrane protein, amyloid precursor protein (APP), by sequential β- and γ-secretase cleavage [2]. Several lines of evidence support the hypothesis that Aβ is a key trigger of AD pathogenesis [28]. The vast majority of AD cases appear in late-onset sporadic form. While aging is the greatest environmental risk factor of the sporadic form, the presence of the ε 4 allele in apolipoprotein E (ApoE4) represents the most important genetic risk factor [29]. Rare, familial, early-onset autosomal dominant forms of AD (FAD) are found to involve mutations in the genes encoding APP and presenilins (PS1 and PS2). Presenilins form the catalytic core of the aspartyl protease γ-secretase complex responsible for intramembranous processing of a variety of type I membrane proteins, including APP. Cleavage of APP at the N-terminus by β-secretase and at the C-terminus by the γ-secretase complex constitutes the amyloidogenic pathway, which yields the APP intracellular domain (AICD) fragment in addition to Aβ. In the non-amyloidogenic pathway, α-secretase cleaves APP within the Aβ domain, which results in generation of a soluble fragment of APP (sAPPα) and a membrane-bound carboxyl-terminal fragment, thereby precluding the formation of Aβ (Figure 2). Strikingly, quite a few molecules central to AD have been found to play a regulatory role in aspects of adult neurogenesis. Using a loss-of-function animal model by crossing ApoE-deficient mice to a nestin-GFP reporter, Yang et al. demonstrated that lack of ApoE increased proliferation of early NPCs within the DG, which resulted in depletion of the overall pool of Type 1 NPCs over time [30]. A recent study showed that NPCs infected with lentiviral vectors expressing short interfering RNA (siRNA) for the exclusive knockdown of PS1 in the neurogenic microenvironments exhibited a dramatic enhancement of cell differentiation in a γ-secretase-dependent manner [31]. BrdU incorporation assay has shown that AICD expression decreased hippocampal progenitor cell proliferation and survival [32]. In contrast to the negative effect of AICD on neurogenesis, the sAPPα was shown to protect neurons and promote neurogenesis, possibly mediated by its ability to prevent overactivation of CDK5 and tau hyperphosphorylation [33]. For instance, sAPPα regulated proliferation of EGF-responsive NPCs in the subventricular zone (SVZ) of the lateral ventricle, the other neurogenic area of the adult brain [34]. Although it was suggested that the effect of sAPPα was limited in the SVZ, due to the absence of sAPPα-binding sites in the SGZ [34], this conclusion contrasts with the observation that deficiency of the sortilin-related receptor with type-A repeats resulted in increased proliferation and survival of NPCs in both the SVZ and the DG, most likely as a consequence of an enhancement of local sAPPα production [35]. Information from studies using gene-modified mouse models of AD seems to be more complex (Table 1). Mutations of PS1 generally have a negative effect on the production of new neurons, although the dependency of γ-secretase remains to be confirmed. Using PS1M146V knock-in mice in which the M146V mutation is incorporated into the endogenous mouse PS1 gene, Wang et al. reported that the FAD mutation impaired contextual fear conditioning, which is correlated with reduced adult neurogenesis in the DG [36]. Expression of human mutant PS1P117L in transgenic mice decreased the survival of BrdU-labeled NPCs, thereby leading to fewer new β-III-tubulin-immunoreactive neurons being generated in FAD mutant animals during the 4-week postlabeling period [37]. Enriched environment (EE)-induced proliferation and neuronal differentiation of hippocampal progenitor cells in mice harboring transgenes encoding the FAD-linked human PS1 variant PS1ΔE9 or PS1M146L were significantly impaired, although the impairment was at least in part mediated by soluble factors released from microglia, thus indicating a non-cell-autonomous mechanism underlying PS1 regulation of adult hippocampal neurogenesis [38]. Similarly, the proliferation and survival of NPC were reduced in transgenic mice expressing a chimeric mouse-human (mo-hu) APP-695swe (APPswe) polypeptide, a mutated form of APP that causes early onset FAD [39]. An age-dependent decrease in SGZ proliferation was also observed in mice transgenic for human V717F mutant APP, a model of AD with age-dependent accumulation of Aβ_42_-containing plaques [40]. Furthermore, adult neurogenesis was reduced in mice with knock-out (KO) for ApoE or with knock-in (KI) alleles for human ApoE4. Increased BMP signaling promoted glial differentiation at the expense of neurogenesis in ApoE-KO mice, whereas presynaptic GABAergic input-mediated maturation of newborn neurons was diminished in ApoE4-KI mice because of decreased interneuron survival. Potentiating GABAergic signaling restored neuronal maturation and neurogenesis in ApoE4-KI mice to normal levels [41]. Several additional studies examined hippocampal neurogenesis in transgenic mice coexpressing two or three mutated genes. For example, the double knock-in mice harboring mutant APPswe/PS1ΔE9 exhibited a significant reduction in dentate neuroblasts [42]. Long-term survival of newborn neurons was dramatically diminished in transgenic mice overexpressing the Swedish variant of APP and the exon 9-deleted variant of human PS1 [43]. In particular, a significant reduction in proliferation of NPCs and neuronal differentiation was found to take place in the same transgenic mouse line long before amyloid deposition, and interestingly, hyperphosphorylation of the microtubule-associated protein tau in the neurogenic niches might underlie the impaired neurogenesis [44]. Another study was performed in the triple transgenic mice 3xTg-AD that harbors three mutant genes (APPswe, PS1M146V and tauP301L). Decreased proliferation was found in 3xTg-AD mice; this reduction was directly associated with the presence of Aβ plaques and an increase in the number of Aβ-containing neurons in the hippocampus [45]. Despite this compelling evidence for a generalized decrease in new neuron generation in AD mouse models, conflicting observations have been reported. PDGF-APPswe, ind mice (J20), which express the Swedish and Indiana APP mutations driven by a PDGF promoter, showed increased incorporation of BrdU and expression of immature neuronal markers in the DG. These changes occurred before neuronal loss and amyloid deposition could be detected [46]. A separate study revealed an increase in not only NSC proliferation but also neuronal differentiation in J20 mice, which were induced by oligomeric Aβ [47]. Additionally, adult-born DGCs in a similar animal model exhibited longer dendrites, higher spine density and stronger functional responses at an early developmental stage, but they were impaired morphologically and functionally during later maturation. Early inhibition of GABAergic signaling or late facilitation of glutamatergic signaling can normalize neuronal development despite the presence of high Aβ levels, suggesting that abnormal GABAergic neurotransmission or an imbalance between GABAergic and glutamatergic neurotransmission may contribute to impaired neurogenesis in AD models [48]. Notably, although most studies, as described above, have demonstrated either a single compromise or a single enhancement of adult neurogenesis resulting from AD-associated gene mutations, conditional ablation of PS1 in the forebrain and knock out of PS2 in adult mice (PS1/PS2 conditional double knockout) was found to cause neurodegenerative stage-dependent dynamic changes. Cell proliferation was significantly enhanced at early stages of neurodegeneration, whereas the survival of newly generated neurons was impaired up to late stages [49].

**Table 1:** Summary of neurogenesis changes in transgenic mouse models of AD

Genetic Manipulation	Strain Name	Genotype	Promoter	Age (mon)	Pathology (Aβ deposition)	Neurogenesis assessment	Effect	Reference
Knock-in		mo PS1M146V/-		3		BrdU injection twice daily (2 hr apart) for 4 consecutive days	Decreased proliferation and differentiation	[36]
		hu ApoE4		3, 6-7, 12-13		BrdU injection twice (6 hr apart)	Diminished neuronal maturation	[41]
		mo APPswe/PS1ΔE9		8-9 18-24	post	DCX, MCM2 immunostaining	Decreased proliferation	[42]
Transgenic		hu PS1P117L	NSE	3-4		one BrdU injection per day for 12 consecutive days	Decreased survival	[37]
		hu PS1ΔE9, hu PS1M146L	PrP	3		single injection of BrdU	Decreased proliferation and differentiation	[38]
	Tg2576	chimeric mo-hu APPswe	PrP	12-14	pre	five daily injections of BrdU	Decreased proliferation, survival and differentiation	[39]
	PDAPP	hu APPind	PDGF	2 12	pre post	one i.p. injection of BrdU	Decreased proliferation and survival	[40]
	APP/PS1	chimeric mo-hu APPswe/hu PS1ΔE9	PrP	6	pre, post	BrdU injection once daily for 12 consecutive days	Decreased survival	[43]
	APP/PS1	chimeric mo-hu APPswe/hu PS1ΔE9	PrP	2	pre	BrdU injection every 12 hr for 3 days	Decreased proliferation and differentiation	[44]
	3xTg-AD	hu APPswe/PS1M146V/tauP301L	Thy-1.2	4, 9			Decreased proliferation	[45]
	J20	hu APPswe, ind	PDGF	3, 12	pre, post	BrdU injection twice daily (8 hr apart) for 3 consecutive days	Increased proliferation and differentiation	[46]
	J20	hu APPswe, ind	PDGF	3, 5, 9, 11	pre, post	daily injection of BrdU for 5 days	Increased proliferation and differentiation	[47]
	J20	hu APPswe, ind	PDGF	2-3	pre	BrdU injection for 3 days	Accelarated early development but impaired late maturation of newborn neurons	[48]
Knock-out		ApoE		3, 6-7, 12-13		BrdU injection twice (6 hr apart)	Reduced neurogenesis but increased astrogenesis	[41]
		PS1/PS2 forebrain KO		7-9 18-20		single dose injection of BrdU	Enhanced cell proliferation at early stages of neurodegeneration but impaired survival at late stages	[49]

In summary, studies using transgenic models of AD over the past few years have generated mounting evidence supporting alterations in neurogenesis. Because of differences in many parameters, there are apparent discrepancies in the current literature, with a majority of studies reporting compromised neurogenesis and some others observing increased new neuron generation. Thus, a systematic comparison of transgenic mice using the same age, gender, genetic background, neuropathology stage and a similar method of neurogenic analysis (BrdU regimen, cell markers, etc.) would be necessary to draw a definite conclusion. In particular, as promoters determine the expression and neuronal populations of the transgenes, transgenic lines are likely to express distinct levels and/or patterns of AD-associated proteins. Therefore, investigation of different AD transgenic models under the same promoter will be required as well and knock-in lines are more relevant in this regard. A thorough understanding of the molecular mechanisms underlying changes in neurogenesis associated with mutations of AD-causing genes will probably be a crucial next step in understanding how they are linked to the general intrinsic and extrinsic factors that have been identified to regulate neurogenesis. In addition, although numerous studies employing diverse paradigms suggest that FAD-linked transgenic mice exhibit impairment in learning and memory (see [50] for a review), it remains unclear whether the malfunction results from decreased hippocampal neurogenesis. In PS1M146V/- mice, impaired contextual fear learning showed correlation with reduced neurogenesis in the DG, whereas both short-term and long-term synaptic plasticity in area CA1 and DG remained unaffected [36]. This observation has provided thus far the only piece of evidence directly supporting the hypothesis that impaired adult neurogenesis exacerbates memory deficits. Nevertheless, the functional implications of aberrant neurogenesis in transgenic models of AD need to be further characterized to establish the causal relationship.

The existing transgenic animal models only exhibit partial AD pathology and it is thus necessary to correlate the mouse model studies with human samples. One study that analyzed neurogenesis in postmortem human brains by Western blot and immunohistochemistry methods reported that the hippocampus of senile AD patients showed increased expression of immature neuronal marker proteins that signal the birth of new neurons, including DCX, polysialylated nerve cell adhesion molecule, neurogenic differentiation factor and TUC-4. The authors hypothesized that facilitation of neurogenesis might be a self-compensating mechanism to replace the lost neurons and that stimulating hippocampal neurogenesis might provide a new treatment strategy for AD [51]. However, immunostaining for Ki-67, GFAP and DCX in presenile AD cases revealed no indications of altered DG neurogenesis [52]. Moreover, Aβ-associated increases in BMP6 expression were found accompanied by reduced markers of neurogenesis in the hippocampus of human patients with AD [53]. Obviously, these findings are not in agreement, and the discrepancies may arise from stage of the disease, treatment provided to the patients and method for labeling proliferating cells. More systematic studies are required for further clarification.

## Environmental Enrichment, Neurogenesis and AD

It has become increasingly evident that neurogenesis in the adult brain is under the influence of key molecules underlying AD and the alterations take place early in life. Thus, the process of new neuron addition may have a reciprocal connection with AD pathogenesis rather than be simply a result of neural dysfunction, a possibility that opens new opportunities for cellular therapy for this disease. Physical exercise or exposure to EE, a putative positive regulator of adult neurogenesis [11], improves cognitive performance in transgenic mouse models of AD [54-56]. Complex environmental experience has been reported to reduce Aβ levels and amyloid deposition, rescue impaired neurogenesis, and significantly enhance hippocampal LTP in APPswe/PS1DE9 mice [57,58]. Both running and EE increase the number of newborn granule cells in the DG of APP23 mice and improve their water maze performance [59,60]. However, EE does not enhance neurogenesis in transgenic mice harboring FAD-linked PS1 variants [38] or in forebrain-specific PS1 knockout mice [61], and it even suppresses neurogenesis in ApoE4 transgenic mice [62]. Taken together, these studies indicate that the effects of exercise and EE on adult neurogenesis vary between the mouse models of AD. Future experiments should investigate their impact on transgenic mice exhibiting different aspects of AD pathology and determine the dependence of observed behavioral improvement on rescued neurogenesis.

## Conclusions

We have presented evidence that multiple molecular players known to contribute to AD pathogenesis, mainly ApoE, PS1, APP and its metabolites, can modulate adult hippocampal neurogenesis. The apparent discrepancies between increased and decreased new neuron production in studies on human and transgenic murine models might be reconciled by a thorough investigation of different NSC populations under different disease conditions. Notably, alterations in neurogenesis occur at the very early stage of AD progression, prior to processes that may secondarily affect neurogenesis, such as neuronal loss, amyloid deposition and inflammation. These findings suggest that neurogenesis represents an integral part of AD pathology. On the one hand, expression of AD-causing molecules impinges upon neurogenesis and dysregulated neurogenesis might in turn facilitate disease progression. On the other hand, conditions that stimulate endogenous neurogenesis (e.g., environmental stimuli, physical activity, trophic factors, cytokines, and drugs) may help to promote the regenerative and recovery process. Although newly generated DGCs are very small in number as compared to the degenerating neurons in AD, and therefore unlikely to achieve global repair, it is possible to slow down or prevent severe cognitive decline by eliminating initial deleterious triggers. Furthermore, as compared to classical neuropathological hallmarks of AD, adult neurogenesis may serve as an early-stage disease model for drug testing and screening. The development of noninvasive detection of adult neurogenesis and specific biomarkers might provide new means of early diagnosis of AD.

## List of abbreviations used

Aβ: amyloid-β; AD: Alzheimer's disease; AICD: APP intracellular domain; APP: amyloid precursor protein; BrdU: bromodeoxyuridine; DCX: doublecortin; DG: dentate gyrus; DGC: dentate granule cell; EC: entorhinal cortex; EE: enriched environment; FAD: Familial Alzheimer's disease; LTP: long-term potentiation; NPC: neural progenitor cell; NSC: neural stem cell; PS: presenilin; sAPPα: soluble amyloid precursor protein alpha; SGZ: subgranular zone; SVZ: subventricular zone; 3xTg-AD: triple transgenic mice.

## Competing interests

The authors declare that they have no competing interests.

## Authors' contributions

Manuscript drafted by YM and edited by FHG. All authors read and approved the final manuscript.
